# Structural Carotid Atherosclerosis and Mild Behavioral Impairment: A Cross‐Sectional Study in a Community‐Dwelling Population

**DOI:** 10.1002/kjm2.70297

**Published:** 2026-09-24

**Authors:** Po‐Han Chen, Yi‐Ya Fang, Yen‐Ju Lin, Mei‐Feng Huang, Yi‐Chun Yeh, Hsiu‐Fen Lin, Cheng‐Sheng Chen

**Affiliations:** ^1^ Department of Psychiatry Kaohsiung Medical University Gangshan Hospital, Kaohsiung Medical University Kaohsiung Taiwan; ^2^ Department of Psychiatry Kaohsiung Medical University Hospital, Kaohsiung Medical University Kaohsiung City Taiwan; ^3^ Graduate Institute of Clinical Medicine College of Medicine, Kaohsiung Medical University Kaohsiung City Taiwan; ^4^ Graduate Institute of Medicine College of Medicine, Kaohsiung Medical University Kaohsiung City Taiwan; ^5^ Department of Psychiatry, School of Medicine, College of Medicine Kaohsiung Medical University Kaohsiung City Taiwan; ^6^ Department of Neurology Kaohsiung Medical University Hospital, Kaohsiung Medical University Kaohsiung City Taiwan; ^7^ Department of Neurology, School of Medicine, College of Medicine Kaohsiung Medical University Kaohsiung City Taiwan

**Keywords:** carotid atherosclerosis, intima–media thickness, mild behavioral impairment, vascular pathology

## Abstract

Mild behavioral impairment (MBI) is a late‐life neuropsychiatric syndrome proposed as an early clinical manifestation of neurodegenerative disease. Vascular pathology contributes to late‐life cognitive and behavioral changes; however, its relationship with MBI remains insufficiently characterized. This cross‐sectional study investigated whether structural carotid atherosclerosis is associated with MBI and its symptom domains in 563 community‐dwelling, dementia‐free adults aged ≥ 50 from the Kaohsiung Atherosclerosis Longitudinal Study between 2018 and 2021. MBI was assessed using the informant‐rated Mild Behavioral Impairment Checklist, evaluating apathy/motivation, affective dysregulation, impulse dyscontrol, social inappropriateness, and abnormal thought/perception. Structural carotid atherosclerosis was evaluated by ultrasound, assessing plaques and mean carotid intima–media thickness (cIMT) across the common carotid artery (CCA), bifurcation, and internal carotid artery. Advanced structural atherosclerosis was defined as the presence of plaque or top‐quartile cIMT. Multivariable regression models examined associations between carotid atherosclerosis and MBI severity, adjusting for demographics, medical conditions, and global cognition. Advanced structural atherosclerosis was independently associated with higher MBI scores (*β* = 0.143, *p* = 0.001). Elevated cIMT—but not plaque—was significantly associated with MBI severity. Segment‐specific analyses showed increased CCA IMT was the only vascular marker significantly related to MBI total scores. Furthermore, advanced structural atherosclerosis was associated with increased odds of decreased motivation, impulse dyscontrol, and social inappropriateness. In conclusion, structural carotid atherosclerosis, particularly elevated CCA IMT, is associated with greater MBI symptom burden in middle‐to‐older adults. Subclinical vascular pathology may contribute to early neurobehavioral changes, highlighting the importance of vascular health in identifying individuals at risk for neurodegenerative disorders.

## 1 Introduction

The global rise in population aging has intensified the public health importance of early detection strategies for dementia and related neurodegenerative disorders. As the number of older adults increases worldwide, identifying individuals at risk before the onset of overt cognitive impairment and functional decline has become a central focus of contemporary dementia prevention frameworks [1, 2]. An early cohort study has observed behavioral symptoms emerge few years prior to the diagnosis of dementia, suggesting their potential value as early clinical indicators [3]. Traditional approaches that rely on cognitive markers may miss early neurobiological changes, highlighting the need for novel behavioral indicators capable of capturing prodromal disease states.

Mild behavioral impairment (MBI) has emerged as one such promising construct. Conceptualized as a later‐life neuropsychiatric syndrome characterized by the de novo and persistent emergence of changes in behavior, personality, affect, or motivation, MBI is proposed to represent an early manifestation of neurodegenerative disease before measurable cognitive deficits appear [4]. Increasing evidence indicates that MBI may precede and predict later cognitive impairment, progression to mild cognitive impairment, and conversion to dementia, suggesting that subtle neuropsychiatric symptoms may serve as early clinical markers of brain vulnerability [5, 6].

Parallel to neurodegenerative process [7], vascular contributions to late‐life cognitive and neuropsychiatric outcomes have been increasingly recognized. Cerebrovascular disease, small vessel pathology, and related structural changes are well‐established determinants of cognitive decline, depressive symptoms, and dementia [8, 9, 10]. Such vascular pathology often coexists with neurodegenerative processes, creating mixed‐pathology states that may exacerbate clinical expression and accelerate disease progression. Understanding these vascular–neuropsychiatric relationships may thus illuminate modifiable pathways relevant for dementia prevention. Carotid ultrasound is a noninvasive and community‐feasible tool for assessing cerebrovascular health. Structural carotid atherosclerosis, commonly evaluated using carotid plaques and carotid intima–media thickness (IMT), is commonly used to evaluate levels of structural carotid atherosclerosis and cerebrovascular risk. Carotid plaques and elevated IMT have been consistently associated with increased risk of stroke, cardiovascular events, cognitive impairment, and dementia in both clinical and population‐based cohorts [11, 12, 13, 14]. These markers reflect both focal and diffuse vascular pathology and may capture downstream small vessel injury, cerebral hypoperfusion, and inflammatory processes that contribute to cognitive and behavioral symptoms.

Despite extensive evidence linking vascular pathology with cognitive and neuropsychiatric outcomes, relatively little is known about whether structural carotid atherosclerosis is associated with MBI. Given that MBI may reflect early neurobiological changes sensitive to vascular injury—and that carotid atherosclerosis is a modifiable risk factor—it is plausible that increased carotid burden may be linked to the emergence of MBI symptoms in later life. However, empirical data on this relationship remain sparse, particularly in community‐based populations without dementia. Clarifying this association may yield important insights into vascular‐behavioral pathways and inform early risk stratification strategies. In this context, the present study investigates the relationship between structural carotid atherosclerosis and MBI in a community‐dwelling population. Structural carotid atherosclerosis in this study is defined as the presence of carotid plaques and increased mean carotid IMT across available ultrasonographic segments. We hypothesized that greater carotid atherosclerotic burden would be associated with higher MBI symptomatology. Furthermore, we examined whether these associations vary across MBI domains and carotid arterial segments.

## 2 Materials and Methods

### 2.1 Study Design and Participants

**FIGURE 1:**
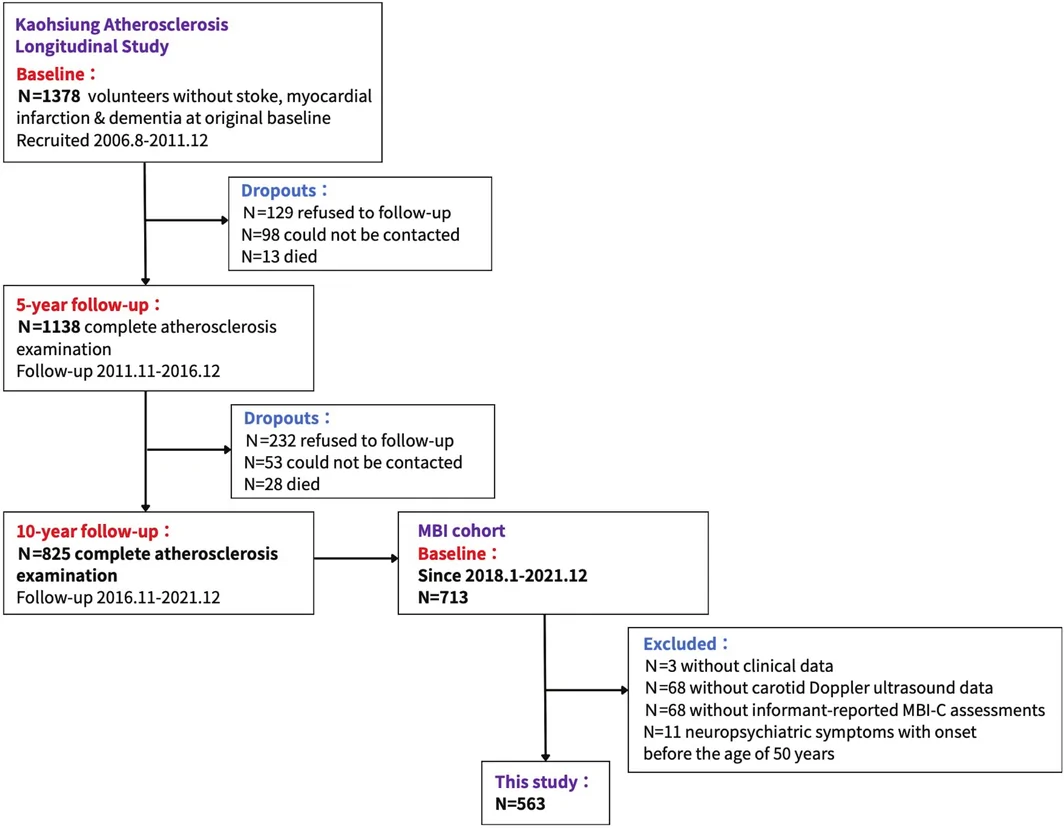
Flowchart of cohort participant.

This research utilized data from the Kaohsiung Atherosclerosis Longitudinal Study (KALS), an established cohort study between 2006 and 2011 [15]. Participants were recruited voluntarily from the community via posters. The study sample consisted of community‐dwelling older adults, aged 50 or above, without a history of stroke, myocardial infarction, dementia, or carotid surgery. Exclusion criteria included existing dementia or severe neuropsychiatric diseases. Figure 1 shows the flow diagram of study enrollment. The original KALS baseline cohort comprised 1378 individuals. Between 2018 and 2021, 713 community‐dwelling middle‐aged and older adults from this cohort were invited and completed the MBI assessment. For this current MBI study, inclusion criteria were age at least 50 years and absence of a dementia diagnosis. Participants who developed incident stroke or myocardial infarction during the follow‐up period were not excluded from the current analysis; rather, these conditions were adjusted for as covariates in our statistical models. Exclusion criteria were prior carotid surgery and major neurological or psychiatric disorders, including epilepsy, Parkinson's disease, schizophrenia, or mood disorders. Participants were excluded for analysis because of missing data, including three without clinical data, 68 without carotid Doppler ultrasound data, and 68 without informant‐reported MBI‐C assessments. An additional 11 participants were excluded because of neuropsychiatric symptoms with onset before the age of 50 years. After these prespecified exclusions, 563 participants were included in the final analyses. The Institutional Review Board of Kaohsiung Medical University approved the protocol (KMUHIRB‐SV(I)‐20160065). All participants provided written informed consent.

### 2.2 Mild Behavioral Impairment

MBI was defined according to the International Society to Advance Alzheimer's Research and Treatment research diagnostic criteria, requiring new and persistent symptoms at or after age 50, lasting at least 6 months, and causing at least mild social or interpersonal impact. The five symptom domains are decreased motivation, affective dysregulation, impulse dyscontrol, social inappropriateness, and abnormal perception or thought content [4, 16]. MBI was assessed using the MBI‐Checklist (MBI‐C), a 34‐item scale that evaluates five major domains: apathy/motivation, affective dysregulation, impulse dyscontrol, social inappropriateness, and abnormal thought/perception. MBI has two versions of the informant‐rated and self‐rated MBI‐C, and in this study, informant‐rated data served as the primary outcome. Scores reflect severity on items endorsed as late‐onset and persistent; domain scores are summed to form the total score.

### 2.3 Carotid Ultrasonography

Carotid ultrasonography was performed on a Philips HD11 system with a broadband linear array transducer by a single experienced sonographer blinded to clinical data. Participants were supine, and anterior oblique insonation was used. Intima–media thickness was measured at the far wall of predefined segments: the common carotid artery (CCA), the bifurcation of the common carotid artery (BIF), and the internal carotid artery (ICA), excluding areas with visible plaques. For each segment, right and left sides were measured when obtainable. The mean carotid IMT (cIMT) was defined as the average across up to six segmental values. Carotid plaque was defined as a focal structure encroaching into the lumen by at least 50% of the adjacent IMT, recorded across 10 standard locations, with images read by a neurologist [17, 18, 19].

### 2.4 Definition of Structural Carotid Atherosclerosis and Quantiles

Structural carotid atherosclerosis was examined in three ways. First, we evaluated an “advanced structural atherosclerosis” composite defined as the presence of carotid plaques and a mean cIMT in the study‐specific top quartile. Second, we examined the two components separately: presence of any plaque or mean cIMT top quartile. Third, we examined segment‐specific IMT top quartiles for the common carotid artery, the bifurcation, and the internal carotid artery. Quartiles were determined from the distribution observed in this study sample.

### 2.5 Covariates and Statistical Analysis

Covariates were selected a priori based on prior literature and included age, sex, education, medical diseases (hypertension, dyslipidemia, diabetes, cancer, myocardial infarction, stroke), cigarette use ever, and global cognition was assessed using the Montreal Cognitive Assessment (MoCA) total score [10, 14, 20, 21, 22].

Analyses were conducted using IBM SPSS Statistics version 20. Continuous variables are reported as means with standard deviations, and categorical variables as counts with percentages. Group comparisons of MBI‐C scores between carotid exposure groups were performed using independent‐samples *t*‐tests. To account for potential confounding, multiple linear regression model examined adjusted differences in MBI‐C scores across groups. Multivariable logistic regression estimated associations between carotid exposures and dichotomous outcomes, including MBI top‐quartile domain scores, adjusting for age, sex, education, medical diseases, cigarette use ever, and MoCA. Two‐sided *p* values less than 0.05 were considered statistically significant.

## 3 Results

### 3.1 Participant Characteristics

A total of 563 participants were analyzed. Carotid plaques were found in 364 participants (64.7%), with an average plaque count of 1.38 (SD = 1.60). The mean cIMT was 0.73 mm (SD = 0.12), and 140 participants (24.9%) were in the highest quartile. Among them, 237 had plaques only, 13 had top‐quartile cIMT only, and 186 had neither. A total of 127 (22.6%) participants had both plaques and top‐quartile cIMT, meeting the criteria of advanced structural atherosclerosis in this study. Table 1 presents the demographic, clinical, and cognitive characteristics of the study population stratified by the presence of advanced structural carotid atherosclerosis. Participants with advanced carotid atherosclerosis were notably older (*t* = 5.15, *p* < 0.001), with a mean age of 68.92 years (SD 6.84), compared with 65.17 years (SD 7.33) in those without advanced disease. Sex distribution also differed substantially between groups (*χ* ^2^ = 19.44, *p* < 0.001): men constituted 59.8% of the advanced atherosclerosis group, compared with 37.8% in the nonadvanced group. Regarding vascular and systemic comorbidities, participants with advanced carotid atherosclerosis consistently exhibited a higher burden of traditional cardiovascular risk factors, including hypertension (*χ* ^2^ = 6.98, *p* = 0.008) and a history of myocardial infarction (*χ* ^2^ = 6.19, *p* = 0.013) but not diabetes mellitus, hyperlipidemia, stroke and cancer. Smoking history also showed a higher proportion among those with advanced carotid atherosclerosis (22.8% vs. 14.0%; *χ* ^2^ = 5.73, *p* = 0.017). Cognitive performance, assessed using the MoCA, revealed subtle but noteworthy differences. Participants with advanced carotid atherosclerosis had slightly lower (*t* = 2.29, *p* = 0.02) mean MoCA scores (25.36, SD 3.19) compared with those without advanced disease (26.04, SD 2.86).

**TABLE 1:** Baseline demographics, clinical data, and features of structural atherosclerosis in study participants (*N* = 563).

	Total (*N* = 563)	Advanced structural carotid atherosclerosis		
		Yes (*N* = 127)	No (*N* = 436)	Yes versus No
	*N* (%)/mean (SD)	*N* (%)/mean (SD)	*N* (%)/mean (SD)	Statistics
Age (years)	66.02 (7.38)	68.92 (SD 6.84)	65.17 (SD 7.33)	*t* = 5.15, *p* < 0.001
Sex (male)	241 (42.8%)	76 (59.8%)	165 (37.8%)	*χ* ^2^ = 19.44, *p* < 0.001
Education (years)	13.49 (2.98)	13.09 (SD 3.37)	13.61 (SD 2.85)	*t* = 1.73, *p* = 0.08
Medical conditions				
Hypertension	231 (41.0%)	65 (51.2%)	166 (38.1%)	*χ* ^2^ = 6.98, *p* = 0.008
Diabetes mellitus	86 (15.3%)	25 (19.7%)	61 (14.0%)	*χ* ^2^ = 2.46, *p* = 0.12
Dyslipidemia	180 (32.0%)	48 (37.8%)	132 (30.3%)	*χ* ^2^ = 2.56, *p* = 0.11
Stroke	13 (2.3%)	2 (1.6%)	11 (2.5%)	*χ* ^2^ = 0.39, *p* = 0.53
MI	14 (2.5%)	7 (5.5%)	7 (1.6%)	*χ* ^2^ = 6.19, *p* = 0.013
Cancer	54 (9.6%)	13 (10.2%)	41 (9.4%)	*χ* ^2^ = 0.08, *p* = 0.78
Cigarettes	90 (16%)	29 (22.8%)	61 (14.0%)	*χ* ^2^ = 5.73, *p* = 0.017
Carotid IMT	0.73 (SD 0.12)	0.89 (SD 0.10)	0.68 (SD 0.07)	*t* = 25.93, *p* < 0.001
CCA IMT	0.77 (SD 0.15)	0.93 (SD 0.17)	0.72 (SD 0.11)	*t* = 16.96, *p* < 0.001
ICA IMT	0.60 (SD 0.14)	0.73 (SD 0.18)	0.56 (SD 0.10)	*t* = 14.27, *p* < 0.001
Bif IMT	0.83 (SD 0.16)	1.02 (SD 0.15)	0.77 (SD 0.11)	*t* = 19.99, *p* < 0.001
Plaque	364 (64.7%)	127 (100%)	237 (54.4%)	*χ* ^2^ = 89.66, *p* < 0.001
MoCA total scores	25.89 (2.95)	25.36 (SD 3.19)	26.04 (SD 2.86)	*t* = 2.29, *p* = 0.022
MBI‐C total scores	2.38 (4.61)	3.65 (SD 5.26)	2.01 (SD 4.34)	*t* = 3.55, *p* < 0.001
MBI‐C domain				
Motivation ≥ 1	112 (19.9%)	37 (29.1%)	75 (17.2%)	*χ* ^2^ = 8.79, *p* = 0.003
Affect ≥ 1	136 (24.2%)	37 (29.1%)	99 (22.7%)	*χ* ^2^ = 2.22, *p* = 0.14
Impulse ≥ 1	191 (33.9%)	62 (48.8%)	129 (29.6%)	*χ* ^2^ = 16.23, *p* < 0.001
Social ≥ 1	70 (12.4%)	24 (18.9%)	46 (10.6%)	*χ* ^2^ = 6.29, *p* = 0.012
Psychosis ≥ 1	42 (7.5%)	13 (10.2%)	29 (6.7%)	*χ* ^2^ = 1.83, *p* = 0.18

*Note:* Continuous variables were analyzed using independent‐samples *t*‐tests. Categorical variables were analyzed using Pearson's chi‐squared tests.

Abbreviations: BIF, bifurcation of common carotid artery; CCA, common carotid artery; ICA, internal carotid artery; IMT, intima–media thickness; MBI‐C, mild behavioral impairment‐checklist; MI, myocardial infarction; MoCA, Montreal cognitive assessment.

Table 2 presents the results of the multivariable linear regression analysis examining factors associated with MBI‐C total scores. Among demographic variables and medical conditions, education demonstrated a statistically significant positive association with MBI‐C total scores (*B* = 0.17, SE = 0.07, *β* = 0.107, *p* = 0.026), which suggests that individuals with higher educational attainment reported or were reported to have higher MBI‐C scores. After controlling for demographic and medical conditions, cognitive performance, as measured by total MoCA scores, was significantly and inversely associated with MBI‐C total scores (*B* = −0.17, SE = 0.08, *β* = −0.112, *p* = 0.027). Lower global cognitive performance was associated with higher MBI symptom burden. Notably, advanced structural atherosclerosis emerged as the strongest independent factor of MBI‐C total scores in the model. Participants with advanced structural atherosclerosis had significantly higher MBI‐C scores (*B* = 1.57, SE = 0.48, *β* = 0.143, *p* = 0.001), even after adjustment for traditional vascular risk factors and cognitive performance. Overall, the model explained a modest proportion of variance in MBI‐C total scores (*R* ^2^ = 0.044; adjusted *R* ^2^ = 0.021). To ensure that prior clinical stroke did not confound these findings, a sensitivity analysis was performed excluding the 13 participants with a history of stroke. The independent association between advanced structural atherosclerosis and higher MBI‐C total scores remained robust and statistically significant (*B* = 1.50, SE = 0.49, *β* = 0.136, *p* = 0.002), indicating that the relationship is primarily driven by subclinical vascular burden.

**TABLE 2:** Factors affecting MBI‐C total scores using a multiple linear regression model (*N* = 563).

	B (SE)	Beta	*p*
Constant	5.02 (3.0)		0.097
Age (years)	−0.01 (0.03)	−0.013	0.783
Sex (male)	−0.37 (0.47)	−0.040	0.433
Education (years)	0.17 (0.07)	0.107	0.027
Medical conditions			
Hypertension	−0.19 (0.43)	−0.020	0.664
Diabetes mellitus	0.16 (0.55)	0.012	0.773
Dyslipidemia	−0.14 (0.43)	−0.014	0.747
Stroke	−0.29 (1.30)	−0.009	0.825
MI	−1.13 (1.27)	−0.038	0.375
Cancer	0.92 (0.66)	0.059	0.168
Substance			
Alcohol	−0.13 (0.56)	−0.011	0.813
Cigarettes	0.46 (0.59)	0.037	0.437
MoCA total scores	−0.17 (0.08)	−0.110	0.029
Advanced structural atherosclerosis	1.57 (0.48)	0.143	0.001
*R* ^2^ = 0.044, adjusted *R* ^2^ = 0.021			

Abbreviations: MBI‐C, mild behavioral impairment‐checklist; MI, myocardial infarction; MoCA, Montreal cognitive assessment.

To examine the association of segmental‐specific IMT or plaque alone with MBI‐C total score, we further conducted multiple linear regression models by putting three segmental IMT quartile or plaque one by one in each model. The results showed only CCA IMT top quartile has significant association with MBI‐C total score (*B* = 1.05, SE = 0.48, *β* = 0.098, *p* = 0.028), but not for ICA IMT top quartile (*B* = −0.71, SE = 0.47, *β* = −0.066, *p* = 0.13), Bif IMT top quartile (*B* = 0.83, SE = 0.46, *β* = 0.078, *p* = 0.074) and with plaque (*B* = 0.06, SE = 0.43, *β* = 0.006, *p* = 0.889).

**FIGURE 2:**
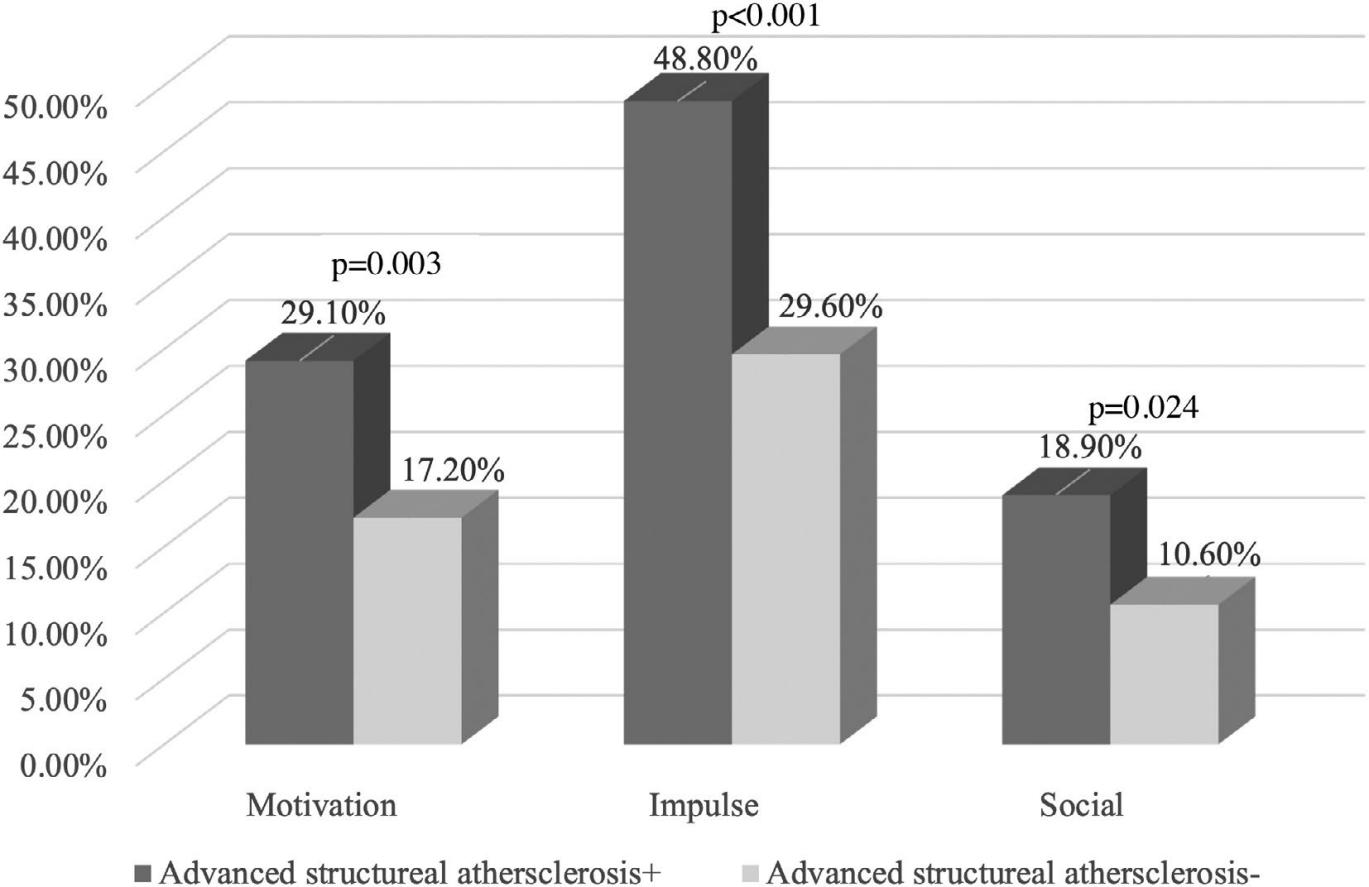
Associations between advanced structural carotid atherosclerosis and specific MBI symptom domains (*N* = 563). MBI, mild behavioral impairment. Data were analyzed using multivariable logistic regression.

As shown in Table 1, motivation, impulse, and social domain were associated with advanced structural atherosclerosis. We further conducted three separate logistic regression models to examine the odds ratios for each domain of advanced structural atherosclerosis by adjusting for demographic data and medical conditions (Figure 2). The results revealed that advanced structural atherosclerosis was significantly associated with the motivation domain (Wald *χ* ^2^ = 8.64, df = 1, *p* = 0.003), corresponding to an adjusted odds ratio (OR) of 2.06 (95% CI: 1.27–3.33), with the impulse domain (Wald *χ* ^2^ = 14.23, df = 1, *p* < 0.001; OR = 2.27, 95% CI: 1.48–3.48), and with the social domain (Wald *χ* ^2^ = 5.07, df = 1, *p* = 0.024; OR = 1.93, 95% CI: 1.09–3.41).

## 4 Discussion

In this community‐based, dementia‐free cohort, we investigated the association between structural carotid atherosclerosis and MBI. Our findings demonstrate that advanced structural carotid atherosclerosis is independently associated with greater overall MBI symptom burden, even after adjustment for demographic characteristics, medical conditions, and global cognitive performance. Notably, among the components of carotid atherosclerosis, carotid IMT—rather than carotid plaque—showed the most consistent association with MBI, particularly when measured at the CCA. Furthermore, this association was domain‐specific, with decreased motivation, impulse dyscontrol, and social inappropriateness showing the strongest relationships with vascular burden.

While vascular pathology has long been recognized as a contributor to cognitive decline and dementia, its role in late‐life neuropsychiatric syndromes has been less well characterized. Previous work has demonstrated associations between cerebrovascular disease and depressive symptoms, apathy, and executive dysfunction, particularly in older adults with small vessel disease or white matter hyperintensities [10, 20, 23]. Our study extends this literature by linking a noninvasive marker of extracranial vascular pathology—structural carotid atherosclerosis—to MBI, a construct specifically designed to capture later‐life, persistent behavioral changes of potential neurodegenerative relevance [24, 25, 26]. Importantly, the observed association remained significant after controlling for traditional vascular risk factors and medical diagnoses, suggesting that echo imaging‐based markers of vascular burden may be more sensitive indicators of neurobehavioral vulnerability than clinical history alone. This finding aligns with prior work showing that subclinical vascular measures, such as IMT, better capture cumulative lifetime exposure to vascular injury than dichotomous disease classifications. From a mechanistic perspective, carotid atherosclerosis may reflect diffuse cerebrovascular compromise, chronic cerebral hypoperfusion, endothelial dysfunction, and inflammatory processes. These factors are hypothesized to subtly disrupt fronto‐subcortical circuits involved in motivation, impulse control, and social behavior [27, 28]. However, because this study lacks concurrent neuroimaging, we cannot definitively conclude that structural carotid atherosclerosis directly causes upstream cerebral small vessel disease. It is equally plausible that elevated carotid IMT and early neurobehavioral shifts represent parallel manifestations of systemic vascular aging and early neurodegeneration, rather than a strict causal chain.

One of the key findings of this study is the differential association between IMT and carotid plaque with MBI outcomes. Although carotid plaques were highly prevalent in this older cohort, plaque presence alone was not significantly associated with MBI‐C total scores or domain‐specific outcomes. In contrast, elevated IMT—particularly in the highest quartile—showed a consistent relationship with MBI severity. By utilizing the top quartile to define advanced atherosclerosis, our analysis inherently captures a threshold association rather than a strictly linear one. This suggests that structural vascular aging may need to accumulate and cross a critical severity threshold before its downstream effects—such as chronic hypoperfusion—manifest as clinically detectable behavioral changes. This distinction may reflect fundamental differences in the biological meaning of these two markers. Carotid IMT is widely regarded as a marker of diffuse arterial remodeling and early atherosclerotic change, whereas plaques represent more focal, advanced lesions [10, 13, 20]. IMT has been linked to systemic vascular aging, microvascular dysfunction, and chronic hypoperfusion, all of which may exert subtle but widespread effects on brain networks before the occurrence of overt ischemic events. In contrast, plaques may be more closely related to embolic risk and clinically manifest stroke, which were exclusionary or rare in the present sample [29, 30]. Our findings are therefore consistent with the hypothesis that MBI represents an early neurobehavioral manifestation sensitive to diffuse vascular injury rather than focal vascular events. This may also help explain why IMT—but not plaque—was associated with MBI in a cognitively intact, community‐dwelling population.

Among segment‐specific IMT measures, only IMT in the common carotid artery was significantly associated with MBI‐C total scores. This regional specificity is noteworthy and biologically plausible. The CCA is particularly sensitive to systemic hemodynamic stress and reflects generalized arterial stiffness and vascular aging [29]. Prior studies have shown that CCA IMT is more strongly associated with cerebral blood flow alterations and white matter changes than IMT measured at the bifurcation or internal carotid artery. From a neurobiological standpoint, diffuse reductions in cerebral perfusion and impaired vascular reactivity may preferentially affect frontal and subcortical regions, which are highly vulnerable to hypoperfusion and are critical for behavioral regulation. These regions underlie motivational drive, inhibitory control, and social cognition—domains prominently represented in the MBI construct [31, 32]. In contrast, affective symptoms and psychotic‐like experiences may be more closely linked to limbic or temporoparietal pathology, which may emerge later or be driven more strongly by neurodegenerative processes [33, 34, 35]. The association between CCA IMT and MBI therefore reinforces the concept that vascular aging may preferentially influence behavioral rather than purely cognitive symptoms in the earliest stages of brain vulnerability. The selective vulnerability of these behavioral domains supports the conceptualization of MBI as a heterogeneous syndrome with potentially distinct biological substrates across domains. It also suggests that vascular pathology may contribute preferentially to specific behavioral phenotypes within the MBI spectrum.

Consistent with prior literature, lower global cognitive performance, as measured by the MoCA, was independently associated with higher MBI‐C total scores. This finding reinforces the close relationship between subtle cognitive inefficiency and emerging neuropsychiatric symptoms, even in individuals without clinically diagnosed cognitive impairment [36, 37]. Importantly, the association between carotid atherosclerosis and MBI persisted after adjusting for MoCA scores, suggesting that vascular contributions to behavioral change are not merely mediated by global cognition.

When interpreting these findings, it is important to consider the strength of the association. As observed in our linear regression model (Table 2), the overall variance in continuous MBI‐C total scores explained by the full set of covariates is relatively modest (*R* ^2^ = 0.043). However, when evaluating categorical clinical thresholds, the strength of the association becomes more substantial. As demonstrated in the domain‐specific logistic regression models, advanced structural atherosclerosis roughly doubled the odds of presenting with clinically significant symptoms in the motivation, impulse dyscontrol, and social inappropriateness domains. This suggests that while subclinical structural carotid atherosclerosis is one of many contributing factors to overall neurobehavioral variation, it serves as a robust indicator for identifying individuals who have crossed clinical thresholds within specific behavioral spectrums.

The positive association between education and MBI‐C total scores warrants careful interpretation. Statistically, education acts as a suppressor variable in our analyses; while participants with advanced atherosclerosis trended toward lower educational attainment in unadjusted baseline comparisons, adjusting for global cognition (MoCA) unmasked a positive association between education and MBI‐C scores. Higher educational attainment is traditionally considered protective against cognitive decline through cognitive reserve mechanisms. However, in the context of MBI assessment, it is possible that individuals with higher education may have greater baseline behavioral expectations, leading informants to more readily detect subtle deviations from prior personality or social functioning. Alternatively, higher education may delay cognitive manifestations of brain pathology, allowing behavioral symptoms to emerge as an early signal of underlying disease. This observation highlights the complexity of reserve‐related effects in preclinical neurodegenerative and vascular states.

From a clinical perspective, carotid ultrasonography is noninvasive and widely accessible. Although the present cross‐sectional design cannot establish causality, the consistent association between mean cIMT and MBI, especially at the common carotid artery, suggests that vascular assessment could complement symptom‐based screening when late‐life behavioral change is newly reported. From a research standpoint, longitudinal designs within KALS and other cohorts should evaluate whether increases in cIMT precede incident MBI and whether MBI mediates relationships between vascular aging and cognitive decline. Multimodal approaches that integrate carotid measures with small vessel disease imaging and blood‐based biomarkers may resolve mechanistic pathways linking systemic atherosclerosis, cerebrovascular integrity, and emergent behavioral phenotypes [38, 39, 40]. Strengths include a community‐based sample, use of the noninvasive, widely accessible and standardized carotid ultrasonography with good within‐lab reproducibility, use of validated informant MBI‐C instruments, and comprehensive covariate adjustment. Limitations include the cross‐sectional design, potential reporting bias in informant ratings, lack of detailed plaque morphology, absence of specific carotid hemodynamic parameters which could offer deeper mechanistic insights into downstream cerebral perfusion, and absence of concurrent brain imaging to directly quantify small vessel disease. External validity may be limited by cohort composition, and findings should be replicated in diverse populations.

## 5 Conclusion

In conclusion, this finding underscores the potential role of subclinical cerebrovascular pathology in the development or expression of late‐life neuropsychiatric symptoms. Structural carotid atherosclerosis measured by IMT is associated with MBI in community‐dwelling older adults, with domain‐specific associations and a prominent role for the CCA segment. These findings support a vascular contribution to MBI and highlight the importance of considering cerebrovascular health in the early identification and prevention of neurodegenerative disorders. These results add to converging evidence that vascular pathology contributes to early neuropsychiatric change and support longitudinal, multimodal studies to test causal hypotheses.

## Funding

This work was supported by the National Science and Technology Council (MOST 111‐2314‐B‐037‐037‐MY3) and Kaohsiung Medical University Hospital (KMUH114‐4R70). The funding source had no involvement in study design, collection, analysis, and interpretation of data, writing of the report, and the decision to submit the article for publication.

## Ethics Statement

This study was approved by Kaohsiung Medical University Hospital institutional review board (KMUHIRB‐SV(I)‐20160065).

## Conflicts of Interest

The authors declare no conflicts of interest.

## Data Availability

The data that support the findings of this study are available on request from the corresponding author. The data are not publicly available due to privacy or ethical restrictions.
